# Condom use within non-commercial partnerships of female sex workers in southern India

**DOI:** 10.1186/1471-2458-11-S6-S11

**Published:** 2011-12-29

**Authors:** Kathleen N Deering, Paranita Bhattacharjee, Janet Bradley, Stephen S Moses, Kate Shannon, Souradet Y Shaw, Reynold Washington, Catherine M Lowndes, Marie-Claude Boily, Banadakoppa M Ramesh, S Rajaram, Kaveri Gurav, Michel Alary

**Affiliations:** 1 Division of AIDS, Department of Medicine, Faculty of Medicine, University of British Columbia, Vancouver, Canada; 2 Karnataka Health Promotion Trust, Bangalore, India; 3 URESP, Centre de recherche FRSQ du CHA universitaire de Québec, Québec, Canada; 4 Department of Medical Microbiology, University of Manitoba, Winnipeg, Canada; 5 Department of Community Health Sciences, University of Manitoba, Winnipeg, Canada; 6 BC Centre for Excellence in HIV/AIDS, Faculty of Medicine, University of British Columbia, Vancouver, Canada; 7 HIV and STI Department, Health Protection Services – Colindale, Health Protection Agency, London, UK; 8 Department of Infectious Diseases Epidemiology, Imperial College, London, UK

## Abstract

**Background:**

Although female sex workers (FSWs) report high levels of condom use with commercial sex clients, particularly after targeted HIV preventive interventions have been implemented, condom use is often low with non-commercial partners. There is limited understanding regarding the factors that influence condom use with FSWs’ non-commercial partners, and of how programs can be designed to increase condom use with these partners. The main objectives of this study were therefore to describe FSWs’ self-reported non-commercial partners, along with interpersonal factors characterizing their non-commercial partnerships, and to examine the factors associated with consistent condom use (CCU) within non-commercial partnerships.

**Methods:**

This study used data collected from cross-sectional questionnaires administered to 988 FSWs in four districts in Karnataka state in 2006-07. We used bivariate and multivariable logistic regression analysis to examine the relationship between CCU (i.e., ‘always’ compared to ‘never’, ‘sometimes’ or ‘frequently’) with non-commercial partners of FSWs (including the respondents’ husband or main cohabiting partner [if not married] and their most recent non-paying partner [who is neither a husband nor the main cohabiting partner, and with whom the FSW had sex within the previous year]) and interpersonal factors describing these partnerships, as well as social and environmental factors. Weighting and survey methods were used to account for the cluster sampling design.

**Results:**

Overall, 511 (51.8%) FSWs reported having a husband or cohabiting partner and 247 (23.7%) reported having a non-paying partner. CCU with these partners was low (22.6% and 40.3% respectively). In multivariable analysis, the odds of CCU with FSWs’ husband or cohabiting partner were 1.8-fold higher for FSWs whose partner knew she was a sex worker (adjusted odds ratio [AOR]: 1.84, 95% confidence intervals[CI]: 1.02-3.32) and almost 6-fold higher if the FSW was unmarried (AOR: 5.73, 95%CI: 2.79-11.76]. CCU with FSWs’ non-paying partner decreased by 18% for each one-year increase in the duration of the relationship (AOR: 0.82, 95%CI: 0.68-0.97).

**Conclusions:**

This study revealed important patterns and interpersonal determinants of condom use within non-commercial partnerships of FSWs. Integrated structural and community-driven HIV/STI prevention programs that focus on gender and reduce sex work stigma should be investigated to increase condom use in non-commercial partnerships.

## Background

Information about the non-commercial partners of female sex workers (FSWs) in the context of HIV and other sexually transmitted infection (STI) epidemiology is limited. Of particular interest in HIV/STI prevention and care programming is the observation that, although condom use within the commercial sex partnerships of FSWs is frequently reported to be high, condom use is much lower within non-commercial partnerships [1-5]. HIV preventive interventions targeted toward FSWs have typically focused on increasing FSWs’ condom use with commercial clients, since the contribution of commercial sex partnerships of FSWs and clients to HIV epidemics is believed to be high in many settings [6-9]. Indeed, as suggested in a systematic review of studies in sub-Saharan Africa and Asia, HIV preventive interventions focusing on behaviour change are more effective at increasing condom use within commercial compared to non-commercial partnerships of FSWs [10]. The evidence for increased condom use with non-commercial partners after interventions have been implemented is not conclusive. Some studies show increases in condom use [4,11], while others do not [12-14]. Limited research has been conducted to elucidate the reasons for low condom use within non-commercial partnerships of FSWs and how this can be addressed by HIV programming.

Understanding condom use in non-commercial partnerships is complex. The sex partners of FSWs are usually categorized as commercial/paying versus non-commercial/non-paying. Non-commercial partners can include husbands, boyfriends or lovers, as well “men who have free sex” (e.g., police or others who use power or force) [15]. FSWs have varying degrees of emotional closeness, intimacy or other involvement with these partners, which may influence condom use. Condoms may be used less frequently with non-commercial partners compared to commercial clients in order to make a distinction between work and pleasure [16,17]. Condoms may be preferred in commercial partnerships to create a barrier to intimacy and to gain a sense of control with clients [18]. Not using condoms in non-commercial partnerships can represent positive features of a relationship, such as increased closeness and trust, and so condoms may be avoided to remove a barrier to increased intimacy [18]. Conversely, the use of condoms may also be perceived as a symbol of infidelity and foster mistrust [16]. Fertility desires or the use of other types of contraceptives, including female sterilization may impact whether or not condoms are used in non-commercial partnerships.

Previous research among FSWs in our study setting of southern India has found that exposure to a large-scale HIV preventive intervention (the *Avahan* India AIDS Initiative [19]), while associated with increased condom use with commercial clients, was not associated with increased condom use with FSWs’ non-commercial partners in the first few years of the intervention [20]. The main goal of this study was therefore to explore characteristics of these non-commercial partnerships in more detail to better understand the reasons for consistent condom use (CCU) with non-commercial partners.

## Methods

### Study design and sampling

During 2006-07, in-depth cross-sectional quantitative interviews (Special Behavioural Surveys, SBS) were conducted with 988 FSWs in four districts (Bangalore, Belgaum, Bellary and Mysore) in Karnataka state, southern India. A probability sampling method was employed, using time-location cluster sampling with normalized weights calculated to account for the complex sampling design. Sampling methods were similar to those reported by Ramesh et al [21] for other studies carried out among FSWs in southern India. The SBS collected information on characteristics of two groups of FSWs’ non-commercial partners: (1) their husband (if married) or main cohabiting partner (if unmarried); and (2) their most recent non-paying partner (who was neither a husband nor the main cohabiting partner described above). Only women who reported having sex with their most recent non-paying partner in the year preceding the conduction of the study were included in (2). Of note, these are not mutually exclusive categories and there may be some overlap (i.e., some women with a husband or main cohabiting partner may have an additional non-paying partner). Some women may have a husband, but not necessarily be cohabiting with him (information not available from the survey). The SBS also collected information on social factors, sexual behaviours and condom use with different non-commercial partners as well as commercial partners (i.e., occasional clients, who FSWs are not familiar with and who visit FSWs once; and repeat or regular clients, who FSWs are familiar with and who visit FSWs more than once), as well as on the working environment of FSWs and exposure to the ongoing HIV prevention program [19,22].

### Survey organization and methods

The SBS was implemented by the CHARME-India project in collaboration with the Institute of Population Health and Clinical Research, St John’s Medical College, Bangalore, the Centre hospitalier *afflilié* universitaire de Québec, Québec, Canada and the University of Manitoba, Winnipeg, Canada. The surveys were administered through face-to-face interviews and were conducted anonymously, with no names or personal identifiers recorded. A detailed and standardized consent process was implemented for each respondent. The surveys and their protocols were approved by the Government of India’s Health Ministry Screening Committee, the respective Canadian university ethics boards and St John’s Medical College, Bangalore.

### Outcomes

CCU with each type of partner was derived from the survey question: “How often is a condom used when you have sex with <this partner>?” Women were considered to use condoms consistently with each partner, if they answered ‘always’ compared to inconsistently, which was defined as ‘never’, ‘sometimes’ or ‘frequently’.

### Interpersonal, social and environmental factors

Based on previous literature we defined *a priori* a set of interpersonal factors in the survey specific to each partner that may influence CCU with non-commercial partners [1,23]. Common across the two partner groupings were the following variables: duration of the relationship; number of times had sex with the partner in a month; if the partner asks for anal sex; the partner’s employment status; if the partner knows the respondent is a sex worker; and if the respondent believes her partner has sexual relationships with other women. For women with a husband or cohabiting partner, additional factors explored included: partner’s age; age difference between the husband or cohabiting partner and the respondent; and the number of months stayed together in the past year. For women with a non-paying partner, additional factors included: if the respondent ***ever*** stays or lives with the partner (not necessarily in a formal cohabiting relationship); if the partner provides the respondent with economic support; if the respondent provides the partner with economic support; if the respondent is normally under the influence of alcohol during sex with the partner; and if the partner is normally under the influence of alcohol during sex with the respondent.

For each model of CCU, we also examined the impact of social and environmental factors related to the respondent. Social factors included age, marital status (married versus unmarried, including those FSWs of the *Devadasi* tradition, a form of temple-based sex work whereby women are dedicated through marriage to gods or goddesses [24-26]), age at first sex, age at first sex work and duration of sex work; environmental factors included district of residence, education (literacy), having sex work as sole income, and working environment, which was represented by type of solicitation (independent or through a middleman/pimp) as well as the place of solicitation of clients.

### Statistical analysis

Statistical analysis was conducted using Stata Version 10.1 [27]. Continuous variables were categorized based on previous literature if they did not have a linear relationship with the logit of the binary outcomes [28]. In bivariate analyses, χ^2^ tests were used to assess associations between social and environmental factors, and whether or not FSWs had each type of non-commercial partner, as well as associations between interpersonal, social and environmental factors and CCU. Multivariable logistic regression models were developed with CCU as the outcome, for each of the two types of partners. Inclusion into multivariable models for all potential covariates were based on significance at the P<0.10-level from bivariate analysis. Sampling weights were utilized in multiple regression models to account for the complex sampling design, using survey methods. Multicollinearity in multivariable models was assessed using the variance inflation factor (VIF) and tolerance statistics, corrected for the survey methods employed [29]. Adjusted odds ratios (AORs) and 95% confidence intervals (95% CIs) were reported for multivariable logistic regression. All *P-*values reported are two-sided.

## Results

### Sample characteristics

**Figure 1:**
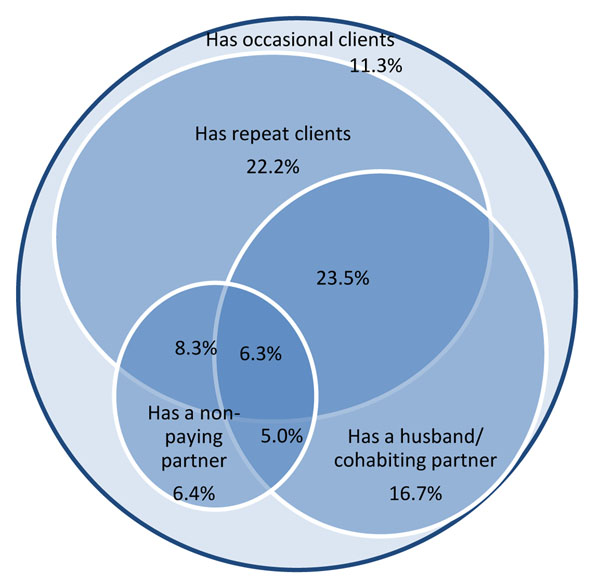
**Sexual partnering patterns of female sex workers across four districts in Karnataka state, southern India**, including women with a husband or cohabiting partner, (at least one) non-paying partner who is neither a husband nor the main cohabiting partner and repeat clients, and who have multiple types of partners. All female sex workers have occasional clients.

Of the total sample of 988 FSWs, 208, 198, 369 and 213 women were recruited in Belgaum, Bellary, Bangalore and Mysore, respectively. The median age was 30 years (interquartile range: 25-35 years) and the median duration of sex work was 5 years (interquartile range: 2-10 years). Of the whole sample, 90.9% of women reported using some form of contraception for family planning (primarily female sterilization or condom use). Overall, 511/985 (51.8%) FSWs reported having a husband or cohabiting partner (with three non-response) and 247/987 (23.7%, with one non-response) reported having a non-paying partner. Of these samples, 506 FSWs had valid responses to condom use with the husband or cohabiting partner and 101 (22.6%) reported CCU with their partner; 247 FSWs had valid responses to condom use with the most recent non-paying partner and 92 (40.3%) reported CCU with their partner. Figure 1 describes the sex partnering patterns of FSWs, according to the types of partners reported by FSWs. All FSWs reported having occasional clients. The highest proportion of FSWs had both a husband or cohabiting partner and repeat clients (23.5%), followed by FSWs with only repeat clients (22.2%) and only a husband or cohabiting partner (16.7%) (Figure 1). The lowest proportion of the population had a husband or cohabiting partner and a non-paying partner (5.0%). Overall, 6.3% of FSWs had all four different types of partners and 11.3% of FSWs only had occasional clients (Figure 1).

Additional file 1 presents characteristics of FSWs according to whether or not they reported having a husband or cohabiting partner, or a recent non-paying partner with whom they had sex within the last year. Of FSWs who reporting having a husband or cohabiting partner 52.8% were currently married and 47.2% were unmarried (i.e., cohabiting). FSWs with and without a husband or cohabiting partner differed significantly in terms of district of residence. Compared to FSWs without a husband or cohabiting partner, FSWs with these partners were significantly more likely to be older when they initiated sex work, be literate, report sex work as their sole income, and have higher CCU with their most recent non-paying partner, with all repeat clients and all occasional clients. FSWs with and without a non-paying partner differed significantly in terms of district of residence. Compared to FSWs who did not have a non-paying partner, FSWs with a non-paying partner were significantly more likely to be younger at first sex, older when they initiated sex work, were less likely to work in public places and more likely to work at home, and had lower CCU with occasional clients.

### Interpersonal characteristics of non-commercial partners and partnerships

Table 1 presents descriptive characteristics of the husband or cohabiting partner of FSWs, and of their most recent non-paying partner, as well as characteristics of these partnerships. The majority of the husbands or cohabiting partners were 35 years or older (mean=36.6 years old). Overall, most of the husbands or cohabiting partners were older than the FSW, with less than 10% being the same age or younger (mean=5.9 years difference). FSWs reported that their relationship with their husband or cohabiting partner had been ongoing for much longer than their relationship with their non-paying partner. In the last year, approximately half of FSWs had stayed in the same place as their husband or cohabiting partner for 9 months or more (mean=7.3 months). Overall, about a quarter of FSWs reported that they had at some time lived or stayed with their non-paying partner. The vast majority of husbands or cohabiting partners and non-paying partners were employed. FSWs reported a higher number of sex acts per month with their husband or cohabiting partner (mean=7.1 sex acts) than they did their non-paying partner (mean=4.6 sex acts). FSWs reported that a higher proportion of their non-paying partners (about 2-fold more) ever asked for anal sex compared with their husbands or cohabiting partners. FSWs reported that a higher proportion of their non-paying partners knew they were sex workers compared to their husband or cohabiting partner (about 1.7-fold more). The majority of FSWs reported that their non-paying partner provided them with some economic support, while the minority of FSWs provided their non-paying partner with economic support. About half of the FSWs reported that they were usually under the influence of alcohol during sexual intercourse with their non-paying partner while slightly more than half reported that their non-paying partner was usually under the influence of alcohol during sexual intercourse.

**Table 1:** Characteristics of non-commercial partners and partnerships of female sex workers in four districts in Karnataka state^1,2^

	Husband or cohabiting partner N=511	Most recent non-paying partner N=247
	Proportion (N)/ median (mean)	

**INTERPERSONAL**		

Partner’s age (years) <35 35 +	38.1% (180) 61.9% (326)	n/a

Age difference FSW older or same age Male partner older (<5 years) Male partner older (5-9 years) Male partner older (10+ years)	9.7% (49) 27.5% (116) 37.0% (208) 25.8% (133)	n/a

Duration of relationship (years)	9 (9.9)	2 (3.8)

Number of months stay together in last year (months) <9 9+	47.9% (215) 57.1% (286)	n/a

Ever have stayed or lived with partner	n/a	27.6% (34)

Partner is employed	94.8 (481)	99.2% (239)

Number of times have sex with partner in a month (times)	5 (7.1)	4 (4.5)

Partner asks for anal sex	16.8 (76)	33.5% (68)

Partner has sexual relationships with other women	41.1 (185)	70.2% (166)

Partner knows respondent is a sex worker	30.8 (147)	49.6% (114)

Partner provides respondent with economic support	n/a	78.3% (185)

Respondent provides partner with economic support	n/a	22.3% (59)

Respondent normally under influence of alcohol during sex w/ partner	n/a	49.6% (119)

Partner normally under influence of alcohol during sex w/ respondent	n/a	63.9% (155)

^1^n/a: question was not available for this type of non-commercial partner

^2^The denominators of each measure may not add up to the total samples due to missing data

### Sexual partnering patterns of non-commercial partners

FSWs reported that almost half of their husbands or cohabiting partners (41.1%) had sex partnerships with other women, while this was the case for the majority of their most recent non-paying partners (70.2%) (Table 1). Respondents whose partner had another sex partner reported that the other partners of their husband or cohabiting partner or non-paying partner could include, respectively, a wife within town (46.6% and 59.8%), a wife outside town (24.9% and 28.9%), a sex worker within town (16.6% and 7.2%), a sex worker outside town (8.2% and 8.8%), another type of female partner within town (6.8% and 5.9%) or another type of female partner outside town (6.7% and 3.9%) (results not shown).

### CCU with non-commercial partners

Table 2 presents results from bivariate analysis examining the relationship between interpersonal, social and environmental factors and CCU, with the two types of non-commercial partners. Compared to FSWs who reported using condoms inconsistently with their husband or cohabiting partner, FSWs who reported using condoms consistently were closer in age to their husband or cohabiting partner, with a shorter relationship duration. A significantly higher proportion also had a husband or cohabiting partner who asked for anal sex and who knew they were sex workers. They were also significantly more likely to be unmarried, literate and older at their age of first sex. They also differed significantly in terms of district of residence and place of solicitation. Compared to FSWs who reported using condoms inconsistently with their non-paying partner, FSWs who reported using condoms consistently had a significantly shorter relationship duration, and were more likely to report at some time living or staying with their non-paying partner. FSWs with CCU also differed significantly in terms of district of residence and place of solicitation from FSWs who used condoms inconsistently.

**Table 2:** **Bivariate relationships between interpersonal, social and environmental factors, and condom use**^1^.

	Husband or cohabiting partner			Most recent non-paying partner		
	
	Consistent condom use N=101	Inconsistent condom use N=405	P	Consistent condom use N=92	Inconsistent condom use N=155	P
			
	Proportion (N)/ Median (Mean)			Proportion (N)/ Median (Mean)		
**Interpersonal**						

Partner’s age (years) <35 35 +	39.0% (35) 61.0% (66)	37.6% (144) 62.4% (260)	0.865			n/a

Age difference FSW older or same age Male partner older (<5 years) Male partner older (5-9 years) Male partner older (10+ years)	6.7% (17) 27.3% (23) 38.9% (36) 27.1% (25)	20.1% (32) 27.6% (92) 30.9% (172) 21.3% (108)	0.002			n/a

Duration of relationship (years)	5 (6.9)	10 (10.7)	<0.001	2 (2.3)	3 (4.6)	<0.001

Number of months stay together in last year (months) <9 9+	57.4% (52) 52.5% (48)	45.1% (163) 54.9% (238)	0.103			n/a

Ever have stayed or lived with partner			n/a	27.5% (18)	10.9% (16)	0.010

Partner is employed	96.9% (4)	94.1% (22)	0.276	100% (0)	99.0% (1)	--

Number of times have sex with partner in a month (times)	4 (6.0)	5 (7.4)	0.194	3 (4.1)	4 (4.7)	0.353

Partner asks for anal sex	26.1% (21)	14.1% (55)	0.017	41.2% (27)	28.3% (41)	0.122

Partner has sexual relationships with other women	39.1% (39)	41.6% (146)	0.755	64.1% (55)	74.5% (114)	0.163

Partner knows respondent is a sex worker	41.5% (42)	27.8% (105)	0.017	46.7% (41)	51.5% (73)	0.553

Partner provides respondent with economic support			n/a	79.9% (71)	77.2% (114)	0.656

Respondent provides partner with economic support			n/a	23.7% (22)	21.3% (37)	0.719

Respondent normally under influence of alcohol during sex with partner			n/a	41.1% (37)	55.3% (82)	0.103

Partner normally under influence of alcohol during sex with respondent			n/a	61.5% (54)	65.5% (101)	0.587

**Social**						

Age <25 25 +	15.6% (11) 84.4% (90)	20.1% (73) 79.9% (332)	0.451	19.2% (20) 80.8% (72)	24.8% (37) 75.2% (118)	0.344

Marital status Currently married Unmarried	19.5% (29) 80.5% (72)	62.3% (269) 37.7% (136)	<0.001	26.2% (28) 73.8% (64)	32.9% (50) 67.1% (105)	0.369

Age at first sex (years) <15 15+	22.2% (26) 77.8% (75)	31.9% (133) 68.1% (272)	0.098	34.4% (27) 65.6% (65)	48.1% (67) 51.9% (88)	0.107

Age at first sex work (years) <20 20+	24.5% (21) 75.5% (80)	23.1% (78) 76.9% (327)	0.809	31.2% (27) 68.8% (65)	40.3% (56) 59.7% (99)	0.268

Duration of sex work (years) <5 5+	39.1% (39) 60.9% (62)	45.0% (196) 55.0% (209)	0.325	39.3% (40) 60.7% (52)	41.1% (70) 58.9% (85)	0.827

**Environmental**						

District Belgaum Bellary Bangalore Mysore	2.9% (3) 13.9% (19) 9.4% (18) 73.8% (61)	16.1% (51) 23.4% (90) 32.7% (173) 27.8% (91)	<0.001	8.8% (9) 27.9% (24) 32.0% (35) 31.3% (24)	51.6% (63) 18.5% (31) 29.5% (60) 0.5% (1)	<0.001

Literate	63.2% (64)	44.7% (194)	0.004	30.5% (31)	22.9% (44)	0.229

Sex work sole income	37.0% (34)	30.1% (108)	0.217	50.5% (49)	63.4% (99)	0.120

Independent solicitation	67.7% (71)	77.2% (307)	0.230	79.4% (75)	76.5% (121)	0.714

Place of solicitation Home Brothel Public places	17.4% (19) 10.9% (9) 71.7% (73)	26.0% (130) 7.4% (45) 66.6% (230)	0.004	26.1% (25) 7.6% (10) 66.4% (57)	29.4% (52) 19.7% (33) 50.9% (70)	0.053

Bivariate relationships between interpersonal, social and environmental factors, and condom use with non-commercial partners of female sex workers in four districts in Karnataka state^1^

^1^n/a: question was not available for this type of non-commercial partner

Table 3 presents results from the multivariable regression modelling analysis. FSWs were 1.8-fold as likely to use condoms consistently with their husband or cohabiting partner if the partner knew they were sex workers (AOR: 1.84, 95%CIs: 1.02-3.32). FSWs who were unmarried (i.e., had a main cohabiting partner rather than a husband or cohabiting partner) were significantly more likely to report using condoms consistently (AOR: 5.73 [2.79-11.76]). CCU with the non-paying partner was significantly associated with a shorter duration of the relationship (AOR: 0.82, 95% CIs: 0.68-0.97). CCU with both types of non-commercial partners was also significantly associated with district in multivariable analysis.

**Table 3:** Multivariable (adjusted) odds ratios (AOR) and 95% confidence intervals (95%CIs)^1,2,3^

	Consistent condom use within different sexual partnerships			
	Husband or cohabiting partner		Most recent non-paying partner	
	
	AOR [95% CIs]	P	AOR [95% CIs]	P

**INTERPERSONAL**				

Age difference FSW older or same age Male partner older (<5 years) Male partner older (5-9 years) Male partner older (10+ years)	0.62 [0.22-1.73] 0.65 [0.24-1.72] 0.56 [0.19-1.63] 1.0 (ref)	0.707 0.778 0.469		n/a

Duration of relationship (years)	1.01 [0.96-1.06]	0.816	0.82 [0.68-0.97]	0.021

Ever have stayed or lived with partner (vs never stayed/lived with partner)		n/a	0.68 [0.13-3.56]	0.644

Partner asks for anal sex (versus partner does not ask for anal sex)	1.32 [0.65-2.66]	0.440	/	/

Partner knows respondent is a sex worker (versus partner does not know respondent is sex worker)	1.84 [1.02-3.32]	0.042	/	/

**SOCIAL**				

Marital status Currently married Unmarried	1.0 (ref) 5.73 [2.79-11.76]	<0.001	/	/

Age at first sex (years) <15 15+	1.0 (ref) 1.12 [0.57-2.21]	0.744	/	/

**ENVIRONMENTAL**				

District Belgaum Bellary Bangalore Mysore	0.06 [0.01-0.28] 0.22 [0.10-0.52] 0.14 [0.06-0.31] 1.0 (ref)	0.029 0.818 0.323	0.03 [0.00-0.05] 0.02 [0.00-0.27] 0.02 [0.00-0.15] 1.0 (ref)	<0.001 0.370 0.025

Literate (versus cannot read or write)	1.56 [0.84-2.89]	0.156	/	/

Place of solicitation Home Brothel Public places	1.13 [0.54-2.36] 1.46 [0.54-3.96] 1.0 (ref)	0.853 0.507	0.89 [0.39-2.01] 0.70 [0.24-2.01] 1.0 (ref)	0.893 0.579

Multivariable (adjusted) odds ratios (AOR) and 95% confidence intervals (95%CIs): Multivariable relationships for the relationship between interpersonal, social and environmental factors and consistent condom use with non-commercial sex partners of female sex workers in four districts in Karnataka state^1,2,3^

^1^n/a: Means that the factor was not available for analysis for that type of non-commercial partner

^2^ The symbol / means that the variable was not significant in bivariate analysis and thus not included in multivariable analysis

^3^Only variables that were significant for one of the two outcomes in bivariate were included in this table for brevity

## Discussion

The findings from this study have helped to elucidate how interpersonal characteristics of partnerships can influence condom use with non-commercial partners of women in sex work in southern India. Overall consistent condom use (CCU) with non-commercial partners was low and FSWs reported lower CCU with their husband or cohabiting partner than with their non-paying partner. Both FSWs and their non-commercial partners were found to be substantially connected to other types of partners through other sex partnerships. FSWs reported that a considerable proportion of these male partners had other sex partners. These partners included wives, FSWs or other types of female partners – both within and outside their local geographic settings (i.e., district of recruitment). These results highlight the vulnerability of FSWs to both acquisition and transmission of HIV/STIs within complex sexual networks, as well as the integral role of FSWs’ non-commercial partners as bridge populations who may facilitate the transmission of HIV to female partners outside the context of sex work.

The longevity of the sexual partnerships with FSWs’ non-paying partner appears to be particularly important in determining CCU, with a longer relationship duration being associated with lower CCU. A more nuanced understanding of what the duration of the relationship represents (e.g. increased trust, closeness or familiarity; decreased decision-making power or control) and how these can be addressed in HIV/STI prevention programming is needed. Although FSWs in southern India are highly economically vulnerable with few comparably well-paying employment prospects [30], factors representing the economic stability of the partner (e.g. employment status of the partner, or whether the partner provided economic support) were not significantly associated with CCU. Because the nature of non-commercial relationships is different from commercial relationships, and the economic support, if it exists, is often non-monetary, the decision to use a condom may be more influenced by interpersonal factors related to relationship intimacy (e.g., trust, emotional closeness, power or reproductive desires) than financial dependence. This is supported by studies of non-commercial partnerships of FSWs in other settings [1,31]. However, economic dependence on the male partner is associated with lower condom use in other settings [23,32] and studies of transactional sex arrangements have suggested that trade-offs within these relationships occur, such as increasing amounts of transfers of support (in terms of money, goods, gifts) in exchange for risky behaviour that is perceived as valuable to the male partner (such as sex without a condom) [23,33], even after adjusting for the duration of the relationship [23]. A better understanding of the type and amount of transfers within non-commercial partnerships of FSWs in southern India, both quantitatively and qualitatively could help to better characterize the influence of economic dependence (or co-dependence) on condom use.

While exposure to interventions has been found to be positively associated with increased condom use by FSWs with their clients, including in our setting [4,12,34-37], [38], condom use within non-commercial partnerships has not been a major focus of most interventions and is rarely directly addressed effectively. Condom use within non-commercial partnerships therefore represents an important intervention point, particularly since many non-commercial partners also have other commercial or non-commercial sex partners. Interventions that include a focus on condom use within non-commercial partnerships of FSWs need to go beyond increasing education and access to address issues of intimacy and trust within relationships from the perspectives of women and men, as well as power disparities that favour the male partner [15,17,39], [40]. This is particularly true in settings where women have lower status than men and reduced economic opportunities, and traditional social norms frame socio-cultural views of condom use [17,41], [42]. Interestingly, although education was associated with higher condom use in bivariate analysis in this study, the effects were removed in multivariable analysis. To better understand the complexity of social and environmental factors influencing condom use in non-commercial partnerships and how to develop interventions to increase condom use, conceptual frameworks that have been useful in explaining behaviour could be employed [43-45]. Factors relating to the intention to act, agency and decision-making power (i.e., perception of behavioural control) of women with respect to condom use should be assessed [46]. Furthermore, interventions must be constructed in ways that acknowledge traditional social norms surrounding condom use [17,41], [42] and the potential role of gender-based violence [39,41] and sexual coercion [41] in lower condom use. Qualitative research methods should be employed to better understand socio-cultural reasons for lower condom use within non-commercial partnerships in this setting.

Interventions designed for clients of FSWs as well as FSWs have been observed to contribute to declines in STI prevalence and increases in condom use [47,48]. However, interventions designed for non-commercial male partners are uncommon, despite the often significant role of non-commercial male partners in determining if condoms are used within these partnerships [1,49]. In India, this is in part because such partners are often hidden or difficult to access [15]. Husbands are particularly inaccessible, since many are not aware of their partner’s involvement in sex work, while other non-commercial partners may be actively involved in the management of their partner’s sex work [15]. Fewer husbands than main cohabiting partners in our study were aware of their partner’s involvement in sex work (23% versus 40%, p=0.01). It may be more effective to design male-focused interventions specifically for other non-commercial partners of FSWs, in this and other study settings. There is some evidence for the success of male-focused interventions which address social norms and gender-based violence in increasing condom use, including in India (the Sonagachi Project) and South Africa (Stepping Stones) [50,51]. Targeting younger males may be particularly effective in terms of changing social norms surrounding sexual behaviour for men and women in India. A systematic review of six couples-focused behavioural interventions (outside the context of sex work) found that involvement in the programs was associated with reduced unprotected sex [52], suggesting that these types of interventions could be beneficial and should be explored. In some areas targeted by the Avahan AIDS Initiative in southern India, an increasing focus of the program has been on increasing safety within non-commercial partnerships of FSWs. Avahan’s primary focus in terms of increasing condom use, however, has been for condom use within commercial partnerships. In this study, condom use with non-commercial partners was statistically significantly different in multivariable analysis according to the district where women were recruited into the study. In particular, condom use was higher in Mysore district compared to the other districts. These differences may be due to differences in program implementation or timing of the intervention implemented in each district. Avahan has been the only intervention in Mysore district, but was not the first (though it is now the only) intervention in other districts. Notably, though, a recent study of three districts in Karnataka state (Mysore, Belgaum and Bellary) found that condom use at last sex with non-commercial (non-marital) partners of FSWs has increased significantly (24.4% to 55.9%) over six years of the intervention [53], although this was not the case earlier in the intervention [12]. The largest increases were observed in Belgaum and Mysore. These results indicate that it may take longer for interventions targeted toward FSWs to have an effect on condom use within non-commercial compared to commercial partnerships, but that positive results can be observed. Additional research should be conducted to better understand the social and structural factors that operate within each district on a macro level to influence condom use, and the intersecting relationship between these factors and intervention impact. Individual-level variables such as those explored in this study may not necessarily capture these influences. Lower condom use with non-commercial relative to commercial partners persists and sustained interventions targeted toward increasing condom use within non-commercial partnerships should continue to be explored and developed. The use of HIV prevention methods that women have more control over (i.e., microbicides, female condom) should be investigated in this study population and within non-commercial partnerships.

In our study, CCU was almost two-fold higher with FSWs’ husband or cohabiting partner when this partner knew the FSW was in sex work. Condom use could be higher in these relationships because of an increased awareness of the risks incurred by these women by the male partner. This could also be due to greater exposure and involvement in HIV/STI programming designed for FSWs, or increased access by HIV/STI programs to male partners. Sex work occupational stigma, which influences women to hide their sex work status from their partners and families, has increasingly been postulated as a major barrier to health access for FSWs [54,55]. These results provide support for sex work being recognized as a more legitimate occupation, where women do not feel obligated to hide their work from their non-commercial partners. However, disclosure of sex work needs to be understood within the context of local socio-cultural views and social norms regarding women’s status and sex work, to help ensure that women’s safety is not compromised by disclosure.

Sentinel surveillance and observational studies suggest that HIV and STIs have decreased among FSWs in Karnataka state since Avahan was introduced [12,56], and mathematical modelling has indicated that the increase in condom use among FSWs with their clients after the intervention was introduced is consistent with decreasing HIV epidemiological trends over 2-3 rounds of survey data collection [57,58]. Although commercial sex partnerships of FSWs play a large role in the spread of HIV [7,59], the lack of information on non-commercial partners makes it difficult to assess their role in the spread of HIV through local sexual networks, and their overall potential contribution to HIV epidemics. However, since condom use remains low in non-commercial partnerships in this setting, to better inform the contribution of non-commercial partnerships to the spread of HIV, future empirical studies should collect information on the sexual behaviour of non-commercial partners with other partners (e.g., numbers and types of other partners, frequency of sexual contacts, condom use) and the presence of HIV infection among non-commercial partners of FSWs. Since almost all information on non-commercial partners has been collected second-hand from FSWs rather than from the perspective of their male partners, studies should be conducted with men.

There are several limitations to this study. The study is based in four districts in Karnataka state, southern India, and may not be generalizable to other regions in India. It is based on self-reported data from cross-sectional surveys, and self-reported data may be subject to social desirability bias [60]. However, our sample size was large, particularly for a marginalized and hidden population of FSWs, and the cluster sampling design was aimed to make the sample as representative as possible. Reported condom use was substantially lower with non-commercial rather than commercial partnerships, indicating that women may have been comfortable reporting higher-risk behaviour with these partners. At the same time, it may be more socially acceptable for women to report lower condom use with non-commercial partners, since women as well as men may associate condom use with infidelity or reduced trust. We were unable to control for fertility desires of respondents, which may affect levels of condom use with non-commercial sex partners [23]. However, since the majority of respondents reported using some kind of birth control for family planning purposes, this indicates that most women were not planning on becoming pregnant. Finally, developing questionnaires grounded in theoretical frameworks previously used in similar populations and settings could be helpful in explaining the reasons for condom use [46].

## Conclusions

The results from this study have revealed important patterns and interpersonal determinants of condom use within non-commercial partnerships of women in sex work. Integrated structural and community-driven sexual and reproductive health and HIV/STI prevention programs that include a focus on gender and reduce social stigma surrounding sex work are needed in settings with high HIV prevalence among FSWs and their non-commercial partners.

## Competing interests

The authors declare that they have no competing interests.

## Authors’ contributions

KND contributed to the conceptual design of the study, conducted the study and the analysis and drafted the manuscript; JB and KS participated in the conceptual design of the study and coordination, made substantial contributions in the interpretation of data; PB, SM, SYS, MCB, CL and MA made substantial contributions in the interpretation of data and critically revised the manuscript for important intellectual content; BMR, KG, SR, RW made substantial contributions to the acquisition and management of the data. All authors read and approved the final manuscript.

## Supplementary Material

Additional file 1 Sample characteristics of social and environmental factors: Sample characteristics according to the type of non-commercial partner of female sex workers (FSWs) in four districts in Karnataka state, including FSWs’ husband or main cohabiting partner or their most recent non-paying partner (who is neither a husband nor the main cohabiting partner). Click here for file
