# Supplementary material for: Condom use within non-commercial partnerships of female sex workers in southern India
Source: BMC Public Health. 2011 Dec 29;11(Suppl 6):S11. doi: 10.1186/1471-2458-11-S6-S11 (PMC3287549; doi:10.1186/1471-2458-11-S6-S11)
Supplement: Additional file 1 — Sample characteristics of social and environmental factors: Sample characteristics according to the type of non-commercial partner of female sex workers (FSWs) in four districts in Karnataka state, including FSWs’ husband or main cohabiting partner or their most recent non-paying partner (who is neither a husband nor the main cohabiting partner). [file 1471-2458-11-S6-S11-S1.doc]

Additional file 1. Sample characteristics of social and environmental factors: Sample characteristics according to the type of non-commercial partner of female sex workers (FSWs) in four districts in Karnataka state, including FSWs’ husband or main cohabiting partner or their most recent non-paying partner (who is neither a husband nor the main cohabiting partner)1

|  | Has a husband or cohabiting partner  N=511 | Does not have a husband or cohabiting partner  N=474 | P | Has a non-paying partner  N=247 | Does not have a non-paying partner  N=740 | P |
| --- | --- | --- | --- | --- | --- | --- |
| **SOCIAL** | | | |  |  | |
| Age  <25  25 + | 19.3% (86)  80.7% (425) | 21.5% (117)  78.5% (357) | 0.565 | 22.5% (57)  77.5% (190) | 19.6% (146)  80.4% (594) | 0.441 |
| Marital status  Currently married  Unmarried | 52.8% (301)  47.2% (201) | 0% (0)  100% (474) | <0.001 | 30.2% (78)  69.8% (169) | 26.4% (222)  73.6% (518) | 0.358 |
| Age at first sex (years)  <15  15+ | 29.7% (161)  79.3% (350) | 42.4% (188)  57.7% (286) | <0.001 | 42.6% (94)  57.4% (153) | 33.5% (255)  66.5% (485) | 0.024 |
| Age at first sex work (years)  <20  20+ | 23.4% (101)  76.6% (410) | 30.6% (159)  69.4% (315) | 0.128 | 36.6% (83)  63.4% (164) | 23.9% (178)  76.1% (562) | 0.003 |
| Duration of sex work (years)  <5  5+ | 43.6% (237)  56.4% (274) | 41.9% (209)  58.1% (265) | 0.653 | 40.4% (110)  59.6% (137) | 43.4% (336)  56.6% (404) | 0.495 |
| **ENVIRONMENTAL** | | | |  |  | |
| District  Belgaum  Bellary  Bangalore  Mysore | 13.3% (56)  21.0% (109)  27.4% (192)  38.3% (154) | 31.8% (152)  17.3% (89)  38.1% (177)  12.8% (56) | <0.001 | 34.3% (72)  22.3% (55)  30.5% (95)  12.9% (25) | 18.4% (136)  18.2% (143)  33.1% (274)  30.3% (187) | <0.001 |
| Literate | 31.4% (172) | 22.6% (111) | 0.034 | 26.0% (75) | 27.4% (208) | 0.739 |
| Sex work sole income | 48.9% (261) | 29.9% (151) | <0.001 | 58.2% (148) | 60.9% (420) | 0.606 |
| Independent solicitation | 74.7% (380) | 78.5% (339) | 0.379 | 77.7% (196) | 76.3% (526) | 0.742 |
| Place of solicitation  Home  Brothel  Public places | 23.8% (149)  8.2% (55)  68.0% (307) | 21.3% (121)  23.9% (135)  54.8% (218) | <0.001 | 28.0% (77)  14.8% (43)  57.2% (127) | 20.8% (193)  16.1% (147)  63.2% (400) | 0.140 |
| **CONSISTENT CONDOM USE** | | | | | | |
| Husband or cohabiting partner | 22.6% (101) |  |  | 23.5% (22) | 22.4% (79) | 0.934 |
| Most recent non-paying partner | 51.0% (52) | 29.9% (40) | 0.010 | 40.3% (92) |  |  |
| Most recent repeat client | 77.3% (209) | 60.5% (187) | 0.045 | 62.3% (81) | 70.6% (314) | 0.381 |
| Occasional clients | 80.1% (388) | 72.7% (343) | 0.040 | 67.1% (156) | 79.6% (577) | 0.005 |

^1^The denominators of each measure may not add up to the total samples due to missing data.
